# Haem-responsive gene transporter enables mobilization of host haem in ticks

**DOI:** 10.1098/rsob.210048

**Published:** 2021-09-01

**Authors:** J. Perner, T. Hatalova, M. Cabello-Donayre, V. Urbanova, D. Sojka, H. Frantova, D. Hartmann, D. Jirsova, J. M. Pérez-Victoria, P. Kopacek

**Affiliations:** ^1^ Institute of Parasitology, Biology Centre of the Czech Academy of Sciences, Ceske Budejovice, Czech Republic; ^2^ Institute of Parasitology and Biomedicine ‘López-Neyra’, CSIC, (IPBLN-CSIC), Granada, Spain

**Keywords:** ticks, HRG, transporter, haem, auxotrophy

## Abstract

Ticks, notorious blood-feeders and disease-vectors, have lost a part of their genetic complement encoding haem biosynthetic enzymes and are, therefore, dependent on the acquisition and distribution of host haem. Solute carrier protein SLC48A1, aka haem-responsive gene 1 protein (HRG1), has been implicated in haem transport, regulating the availability of intracellular haem. HRG1 transporter has been identified in both free-living and parasitic organisms ranging from unicellular kinetoplastids, nematodes, up to vertebrates. However, an HRG1 homologue in the arthropod lineage has not yet been identified. We have identified a single HRG1 homologue in the midgut transcriptome of the tick *Ixodes ricinus,* denoted as *Ir*HRG, and have elucidated its role as a haem transporter. Data from haem biosynthesis-deficient yeast growth assays, systemic RNA interference and the evaluation of gallium protoporphyrin IX-mediated toxicity through tick membrane feeding clearly show that *Ir*HRG is the *bona fide* tetrapyrrole transporter. We argue that during evolution, ticks profited from retaining a functional *hrg1* gene in the genome because its protein product facilitates host haem escort from intracellularly digested haemoglobin, rendering haem bioavailable for a haem-dependent network of enzymes.

## Graphical abstract

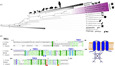

## 1. Introduction

Several parasites have, through evolution, lost genes encoding haem biosynthetic enzymes, yet they retained and perform haem-based metabolism [1]. The parasites therefore need to salvage host haem and distribute it across their cells and body tissues in order to recycle the acquired host haem for their own metabolic demands. Protein components of the molecular network facilitating such haem distribution are, however, poorly understood. Ticks, also, cannot synthesize haem *de novo* and need to operate a regulated acquisition–distribution–disposal network to allow haem-based metabolism and, at the same time, prevent cytotoxicity resulting from haem overload [2]. Upon blood-feeding, engorged tick females acquire and deposit haem into ovaries to allow embryogenesis in laid eggs. The absence of dietary haem leads to the failure of engorged tick females to reproduce [3]. Such maternal haem is acquired as a liberated by-product of lysosomal digestion of host haemoglobin in tick midgut cells [3,4]. A vast excess of haem gets detoxified through intracellular bioaccumulation of haem within so-called residual bodies/haematin granules/haemosomes [5–7], but a certain amount of haem is translocated into the cytosol, where it primarily gets complexed with haem-binding enzymes, chaperons and transporters. The manner in which haem is translocated into the cytosol of tick midgut digest cells is, however, unknown.

Haem is amphipathic in nature and, in the cellular environment, partitions into membranes and the protein pool. Predictions for haem transport range from postulating exclusive proteinaceous transport systems to autonomous diffusion across lipid membranes [8,9]. The former method of transport is mediated by haem transporters, many of which were recently identified in various eukaryotic organisms [10]. Solute carrier protein SLC48A1, known as haem-responsive gene 1 protein (HRG1), has been implicated in such haem transportation in many organisms ranging from unicellular kinetoplastid parasites [11–13], parasitic [14,15] and free-living [16] worms, to mice [17] and humans [18]. However, no homologue has been identified in any species of the arthropod lineage. Here, we have identified the first arthropod HRG1 homologue in the tick *Ixodes ricinus* and demonstrate the conservation of the protein function in haem transportation.

## 2. Results

### 2.1. Phylogenetic analysis underscores the unique conservation of tick HRG1 within arthropods

**Figure 1.**
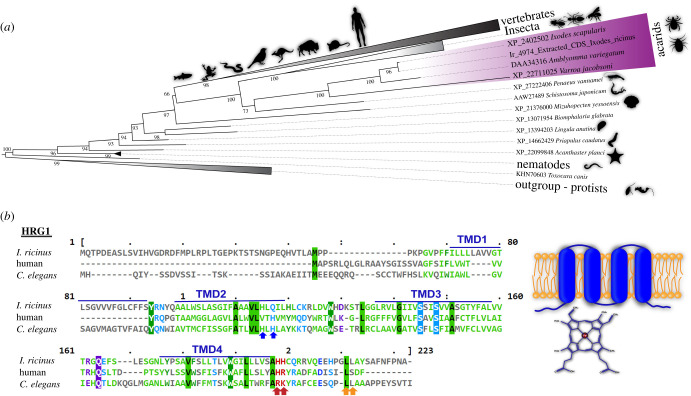
Phylogenetic analysis (*a*) based on HRG1 protein amino acid sequences of vertebrates and invertebrates with a final length of alignment of 143 aa. The tree was computed by the W-IQ-TREE software. Sequences of protists were used as an outgroup to root the final tree. Statistical support for each branch was obtained from the ML bootstrap. (*b*) Amino acid sequence alignment of tick (*I. ricinus*), human and *C. elegans* homologues of *Ir*HRG1. Blue arrows indicate histidine residues implicated in association with haem and red arrows indicate basic amino acid residues of another haem-binding motif [20]. Orange arrows indicate dileucine (D/E)XXXLL motif, a specific sorting signal directing transmembrane proteins to compartments of the endosomal–lysosomal system [16,21–23]. A predicted transmembrane topology of HRG and haem is shown on the cartoon to the right.

The tick HRG1 homologue, *Ir*HRG, was identified (contig ID: Ir-4974; protein ID: JAP70263.1) in our RNAseq data from *I. ricinus* midgut transcriptome analysis [19]. To assess its relationship with HRG1 amino acid sequence data from other organisms, we performed maximum-likelihood (ML)-based phylogenetic analyses of our dataset encompassing blood-feeding nematodes, insects and vertebrates, with protists being used as an outgroup (figure 1*a*). We carried out phylogenetic analyses in three independent runs, where all three runs provided identical results. The final tree was rooted using protist sequences that encode HRG1 transporter [12,13,24]. The individual groups of invertebrates, such as arthropods (insects and acarids), parasitic nematodes, trematodes and molluscs, showed strong support for their clustering. Phylogenetic analyses nested the parasitic nematodes (*Trichinella* sp. and *Toxocara canis*) as the most basal clade in the tree. Species of echinoderms, molluscs, crustaceans and trematodes (*Schistosoma japonicum*) were positioned as a sister taxa to each other. Additionally, these taxa fell on the root of the split for arthropods and vertebrates. The arthropod branch was further divided into acarids and insects, where our protein sequence clustered with HRG data obtained from other acarids. Furthermore, all HRG1 data of other tick species, particularly *Ixodes scapularis* and *Amblyomma variegatum*, were grouped together. The tick-branch had robust support and was separated from the whole acarid group.

Interestingly, the HRG1 homologue has not yet been identified in blood-feeding insect species, but was found to be preserved in several non-blood-feeding insect groups (beetles, termites and ants) (electronic supplementary material, figure S1). HRG1 membrane protein was discovered for all the major groups of vertebrates and seemed to be conserved within each group. Nei's distances, computed by Geneious software, showed the highest level of similarity with the Ixodida data (65.3–97.7%), but, interestingly, a high level of difference within the arthropods (acarids 25.9–32.6%, Insect 18.2–30.1%) (electronic supplementary material, table S1). *Caenorhabditis elegans* HRG1 and its *I. ricinus* homologue contained four transmembrane helices, a glutamic acid—dileucine endo-lysosomal targeting motif, and key histidine residues that protrude through the second transmembrane domain into the endosomal lumen, where they mediate HRG1 activity [20] (figure 1*b*). The ‘FARKY’ key motif of *C. elegans* HRG1 of the endosomal lumen-facing C-terminal is, however, highly mutated in *I. ricinus* HRG1 homologue with an ‘SAHHC’ motif. The duplet of basic amino acid residues within the motif, however, is conserved across all homologues examined: ‘HH’ for *I. ricinus*, ‘HR’ for human and ‘RK’ for *C. elegans* HRG1 (figure 1*b*). As ticks do not encode other likely homologues of *C. elegans hrg* genes, we further denote *I. ricinus* HRG1 homologue as *Ir*HRG.

### 2.2. Expression of *IrHRG* is independent of dietary haemoglobin

**Figure 2.**
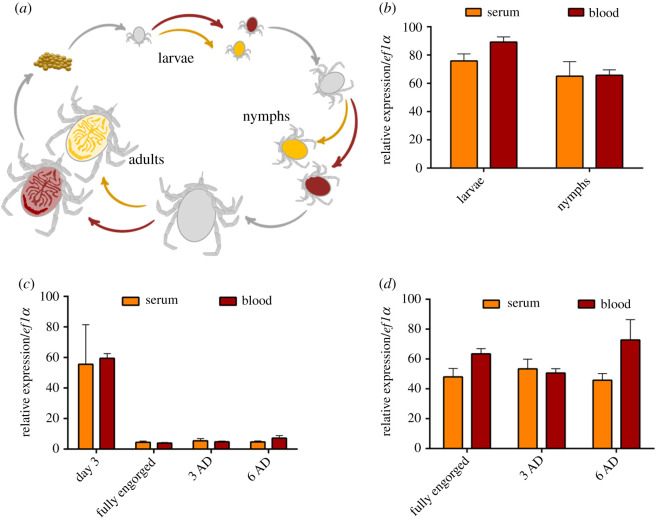
RT-qPCR analysis of *Ir*HRG expression as a function of availability of dietary haem. (*a*) A schematic depiction of blood versus serum feeding of *I. ricinus* developmental stages through an artificial membrane feeding system. (*b*) RT-qPCR analysis of *Ir*HRG in whole-body homogenates from fully engorged larvae and nymphs; each dataset is derived from five independent pools of RNA extracts. (*c*) RT-qPCR analysis of *Ir*HRG in adult female midguts dissected at various time points during and after detachment (AD); each dataset is derived from three independent pools of RNA extracts. (*d*) RT-qPCR analysis of *Ir*HRG in adult female ovaries dissected from fully engorged females and females AD; each dataset is derived from three independent pools of RNA extracts. Means and s.e.m. are shown. In all samples, no *t*-test statistical significance was identified.

Intracellular haem homeostasis in metazoan organisms is facilitated by the haem importer HRG-1 via its transcriptional upregulation at low availability of exogenous haem; hence the name haem-responsive gene (HRG) [16,25]. Our previous transcriptomic study did not indicate any differences in levels of *IrHRG* in midguts of serum- and blood-fed ticks [19]. We re-examined this apparent deviation by RT-qPCR of midguts dissected from ticks fed serum, serum + haemin, serum + haemoglobin or blood, but did not observe any statistically significant differences in levels of *Ir*HRG transcripts (electronic supplementary material, figure S4). To gain a more comprehensive picture of *IrHRG* haem-mediated regulation, we performed a RT-qPCR differential expression analysis of *Ir*HRG in all developmental stages of *I. ricinus* ticks, which were artificially fed either serum or blood (figure 2*a*). In line with our previous transcriptomic data, we did not detect any differential expression of *Ir*HRG in whole-body homogenates of blood- and serum-fed larvae and nymphs (figure 2*b*), nor did we detect differential levels of *Ir*HRG in guts (figure 2*c*) or ovaries (figure 2*d*) dissected from blood- or serum-fed females. These data suggest that the expression of *Ir*HRG transcripts is independent of the presence of haemoglobin haem in the diet.

### 2.3. *Ir*HRG rescues growth of *Δhem1* *S. cerevisiae* by the enhanced import of exogenous haem

**Figure 3.**
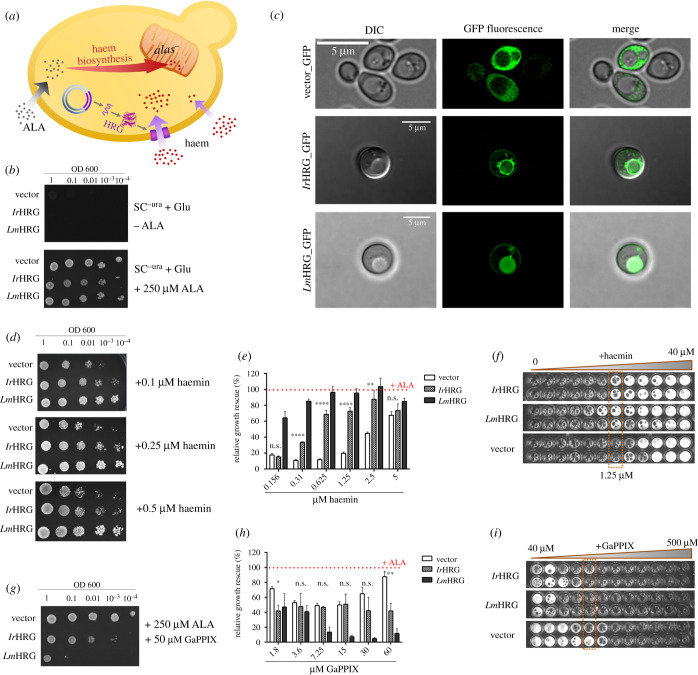
Assessment haem-translocating function of *Ir*HRG in *Δhem1 S. cerevisiae*. (*a*) Haem biology-relevant characteristics are depicted in a drawing of a *hem1Δ* mutant cell lacking the gene for ALA synthase (*alas^−^*). (*b*) Spot growth assay of the haem biosynthesis-deficient yeast strain *Δhem1* in the absence (i) and presence (ii) of exogenous ALA in the culture medium. (*c*) *Ixodes ricinus* HRG heterologously expressed in a haem auxotrophic *S.cerevisiae* strain (*hem1Δ*) as a fused protein with fluorescent GFP (*Ir*HRG_GFP). The figure shows a yeast cell expressing *Ir*HRG_GFP and *Leishmania major* HRG_GFP. A representative cell, with a fluorescence pattern similar to that of the total cellular population, was selected. The vector represents yeast cells transfected with the insert-free plasmid, thus expressing only GFP. (*d–f*) Growth rescue with haem supplementation. (*d*) Growth plates (SC^−ura^ + Glucose) were supplemented with different concentrations of haemin. *Hem1Δ* yeasts transformed with empty plasmid (vector, negative control) or plasmid containing *Ir*HRG and *Lm*HRG were spotted in 10-fold serial dilutions based on optical density at 600 nm. (*e*) Analogous growth rescue assays of these cells in liquid medium supplemented with different concentrations of haemin (158 nM–5 µM) and comparison of the cellular densities to those of yeast supplemented with ALA (defined as 100%); **** indicate *t*-test (vector versus *Ir*HRG) *p* < 0.0001, ** indicate *t*-test (vector versus *Ir*HRG) *p* *=* 0.002, n.s. = not significant. (*f*) A 96-well plate format for estimating catalase activity using detergent-mediated oxygen foaming in a *Δhem1* cell suspension cultured by supplementation with a range of haemin concentrations. (*g,h,i*) Facilitated toxicity with GaPPIX supplementation. The effect of internalization of the toxic haem analogue GaPPIX, through expressed *Ir*HRG, was inspected on plates supplemented with ALA to feed endogenous haem biosynthesis and 50 μM GaPPIX to displace endogenous haem. (*g*) Growth plates (SC^−ura^ + glucose) were supplemented with exogenous ALA and GaPPIX. *Hem1Δ* yeasts transformed with empty plasmid (vector, negative control) or plasmid containing *Ir*HRG and *Lm*HRG were spotted in 10-fold serial dilutions. (*h*) Evaluation of the facilitated toxicity of these cells in liquid medium supplemented with ALA and GaPPIX concentrations (1.8 µM–60 µM). Cellular densities were compared with those of yeast supplemented with ALA only (defined as 100%); ** indicates *t*-test (vector versus *Ir*HRG) *p* = 0.002, * indicates *t*-test (vector versus *Ir*HRG) *p* *=* 0.01, n.s. = not significant. (*i*) A 96-well plate format of catalase activity, estimated using detergent-mediated oxygen foaming in an *Δhem1* cell suspension cultured in medium supplemented with a range of concentrations of GaPPIX.

The absence of haem-mediated regulation of *Ir*HRG levels raised the question whether *Ir*HRG, a distant *Ce*HRG1 and mammalian homologue [16,18], retained its function as a haem transporter. To examine and verify the presumed function of *Ir*HRG as a membrane-localized haem transporter, we used complementation assays with *Saccharomyces cerevisiae* yeasts that do not code for HRG1-related protein homologues [11]. Specifically, we used a genetic mutant yeast *Δhem1 S. cerevisiae* unable to produce δ-aminolevulinic acid (ALA) [12], the first committed precursor of haem biosynthesis. This yeast strain is, therefore, a genetic auxotroph unable to produce endogenous haem and needs ALA for growth (figure 3*a*). The *Δhem1* cells do not grow solely on a sugar source but require exogenous ALA to be internalized and to drive endogenous haem biosynthesis, resulting in uniform cellular densities irrespective of the presence or absence of HRG1 (figure 3*b*). Unlike the wild-type strain, which is not efficient in haem uptake, the *Δhem1* strain exhibited the increased capacity to internalize haem from the culture medium [26]. However, when grown under low haemin supplementation (less than 10 µM) [11,20], this strain struggled to grow unless a haem transporter was ectopically expressed. This serves therefore as a powerful assessment of ectopically expressed haem transporters in order to confirm their predicted function. Such a mutant complementation approach has been employed in numerous previous studies [12,18,20,27]. Using strain *Δhem*1, we also monitored the subcellular localization of *Ir*HRG by expressing it as a fusion protein of *Ir*HRG and C-terminal GFP (*Ir*HRG_GFP). While GFP (empty vector) clearly localized into the yeast cytosol, *Ir*HRG_GFP was predominantely localized to membranes of yeast digestive vacuoles (figure 3*c*), a membrane compartment equivalent to the lysosome of higher eukaryotes; this is consistent with the presence of the dileucine sorting motif in *Ir*HRG (figure 1*b*). *Ir*HRG was also partially localized to the plasma membrane (figure 3*c*). The expression of *Ir*HRG_GFP, monitored by flow cytometry, was identical to that of *Leishmania major* HRG-GFP construct *Lm*HRG_GFP and GFP itself (electronic supplementary material, figure S2). This assumes *Ir*HRG to be expressed to a similar extent as *Lm*HRG_GFP and GFP in the yeast cell, thus permitting a direct comparison of haem transport capacity. To assess the transporter function of *Ir*HRG, a stop-codon containing *Ir*HRG insert was cloned into the plasmid to ectopically produce a non-tagged (no-GFP) *Ir*HRG.

To assess the haem transporting capacity of *Ir*HRG, we evaluated the growth rescue of *Δhem1* cells with low haemin supplementation (0.1–0.5 µM), either on solid agar plates (figure 3*d*) or in liquid culture (figure 3*e*). Indeed, the ectopic expression of *Ir*HRG facilitated enhanced growth of the yeast in a dose-dependent manner corresponding to the haem concentration in the medium, similar to HRG from *L. major* (*Lm*HRG), which was used as a positive control. To assess the bioavailability of haem, we evaluated the activity of catalase, an intracellular haem-dependent enzyme producing oxygen from the decomposition of hydrogen peroxide. When the cellular suspension was supplemented with detergent, foam formation was observed upon the addition of hydrogen peroxide. While empty-vector yeasts produced minute amounts of oxygen foam, *Ir*HRG and control *Lm*HRG producing yeast cells produced substantially higher levels of foam when supplemented with haem or haemoglobin (electronic supplementary material, figure S3). In a dose-dependent assay of twofold dilutions, we noted that *Ir*HRG at least doubled the bioavailability of haem (figure 3*f*).

In a reverse experiment, the *Δhem1* yeast growth medium was supplemented with gallium protoporphyrin IX (GaPPIX), a toxic haemin-related analogue, with coordinated Ga^3+^ ions instead of Fe^3+^ ions in the tetrapyrrole centre [28]. The *Δhem1* yeasts expressing *Ir*HRG/control *Lm*HRG grown in medium supplemented with 50 µM GaPPIX had lower cellular densities than yeasts with no HRG, as evidenced by growth assays on solid (figure 3*g*) and liquid (figure 3*h*) drop-out media. *Ir*HRG thus clearly increased the cellular sensitivity to GaPPIX-mediated toxicity, which was also evident from decreased catalase activity when cells were co-cultured with ALA and GaPPIX (figure 3*i*). These yeast mutant-derived data clearly illustrate the capacity of *Ir*HRG protein to translocate tetrapyrolle molecules and to make them available based on cellular demand.

### 2.4. Blood-feeding confers resistance of ticks to GaPPIX toxicity

**Figure 4.**
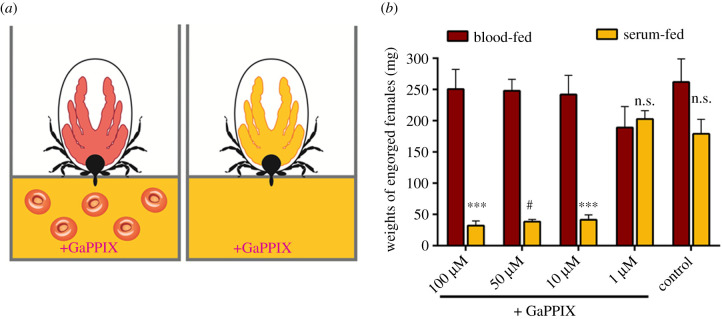
Depletion of dietary haem enables susceptibility of ticks to the toxic porphyrin analogue GaPPIX. (*a*) A schematic depiction of blood- or serum-membrane feeding of ticks, and (*b*) a bar graph showing weights of fully engorged *I. ricinus* females fed blood or serum supplemented with GaPPIX (final concentrations shown). Ticks served blood or serum supplemented with an identical volume of GaPPIX solvent (3.1 µl of 100 mM NaOH) were used as a control. Means and s.e.m. are shown, *n* > 6. ^#^ Indicates *t*-test *p* *=* 0.0001, *** indicates *t*-test *p* *=* 0.001, n.s. = not significant.

To demonstrate the sensitivity of *I. ricinus* ticks to GaPPIX, analogously to *Ir*HRG-expressing yeasts, we designed a differential feeding assay using the artificial feeding system (figure 4*a*). While blood-fed ticks were shown to be insensitive to GaPPIX (figure 4*b*), serum-fed ticks clearly displayed dose-dependent sensitivity to GaPPIX, preventing tick engorgement. Ticks supplied with greater than or equal to 10 µM GaPPIX in dietary serum did not progress through engorgement and achieved negligible weights after 10 days of feeding (figure 4*b*). This observation indicates that blood-feeding prevents GaPPIX-mediated toxicity as GaPPIX is unlikely to displace acquired and complexed haem in endogenous haem-dependent enzymes. GaPPIX complexing with cellular haem-binding enzymes is enabled, however, when no haem is acquired from the tick diet.

### 2.5. RNAi-silencing of *Ir*HRG rescues ticks from GaPPIX-mediated toxicity

**Figure 5.**
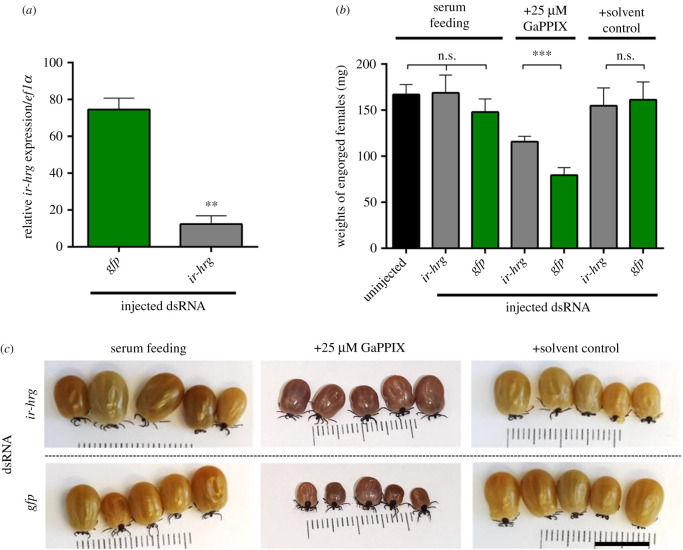
*Ir*HRG knock-down partially rescues GaPPIX-mediated toxicity. (*a*) RT-qPCR analysis of *Ir*HRG in adult female midguts dissected from females fed serum for 3 days. Means and s.e.m. are shown, *n* = 3; ** indicates *p* *=* 0.01. (*b*) Bar graphs show weights of fully engorged ticks that dropped off the membrane between day 7 and day 10. Mean and s.e.m. are shown, *n* ≥ 9, *** indicates *p* *=* 0.001. (*c*) Five representative fully engorged females are shown, the scale bar represent 1 cm.

To verify whether *Ir*HRG plays a role as a porphyrin transporter *in vivo*, we examined whether RNAi-mediated silencing of *Ir*HRG in ticks (*Ir*HRG-KD) can rescue GaPPIX-mediated toxicity in serum-fed ticks. We managed to silence *Ir*HRG transcripts in the midgut of serum-fed *I. ricinus* female ticks down to 16% expression levels of controls (figure 5*a*) and subjected these females to membrane feeding of dietary serum supplemented with 25 µM GaPPIX. We compared the capacity to engorge in *Ir*HRG double-stranded RNA (dsRNA) and *gfp* dsRNA (non-specific dsRNA control) injected ticks. Indeed, *Ir*HRG silencing rescued ticks from GaPPIX toxicity with *Ir*HRG-KD ticks gaining more weight than GFP controls (figure 5*b,c*). To corroborate these data, we followed the uptake of fluorescent metalloporphyrin, zinc protoporphyrin IX (ZnPPIX), in midgut digest cells of the *Ir*HRG-KD ticks. While ZnPPIX was evenly distributed across the tick midgut in control ticks, ZnPPIX accumulated in tick midgut digest cells upon RNAi KD (electronic supplementary material, figure S5). The results of these experiments provide strong evidence that *Ir*HRG is the *bona fide* tetrapyrolle transporter.

### 2.6. RNAi-silencing of *Ir*HRG reduces bioavailability of haem in tick midgut digest cells

**Figure 6.**
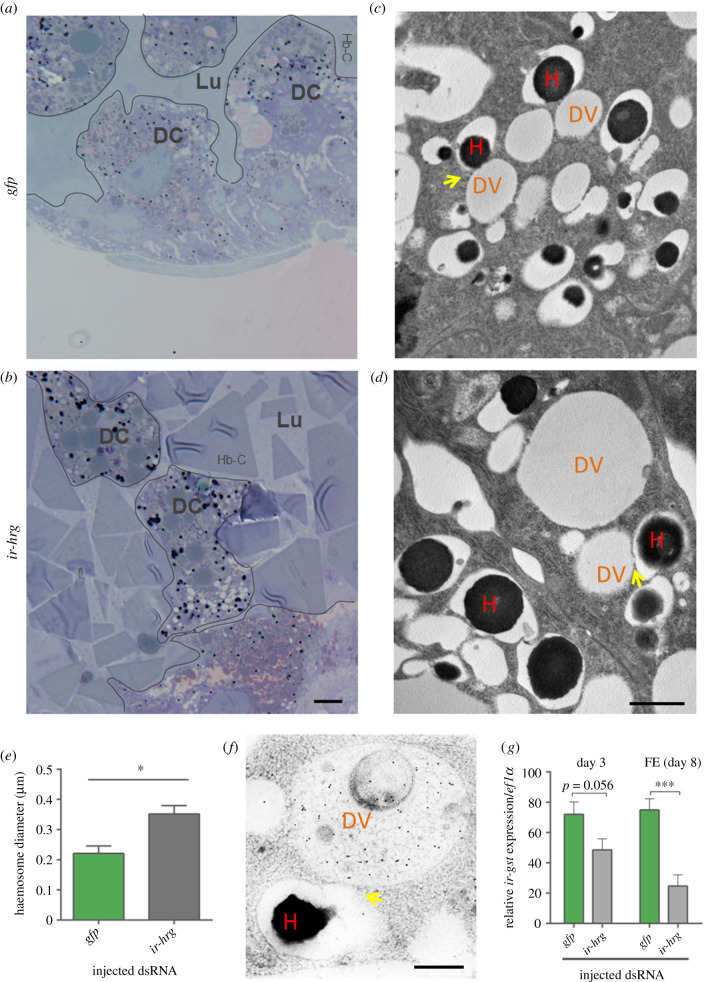
Microscopic examination of haem accumulation in digest cells *Ir*HRG-KD ticks. GFP control (*a,c*) and *Ir*HRG-KD (*b,d*) ticks were partially engorged for 5 days. Ticks were dissected and their midguts embedded, sectioned and visualized by toluidine blue staining under light microscopy (*a,b*) or TEM (*c,d*); DC, digest cell; Lu, midgut lumen; Hb-C, haemoglobin crystals; DV, digestive vesicle; H, haemosomes; yellow arrows point towards contact sites of haemosomes and digestive vesicles. Scale bar (*a*,*b*) indicates 20 µm, scale bar (*c*,*d*) indicates 1 µm. (*e*) Bar graph shows diameters of haemosomes, which were measured by ImageJ from TEM images. Ten haemosomes were measured in five different images of a gut. Each group was represented by three midguts coming from individual ticks. * Indicates *p* < 0.05 of the *t*-test analysed by GraphPad Prism. (*f*) Immuno-gold detection of *I. ricinus* cathepsin B within a midgut digest cell of partially fed adult *I. ricinus* female; DV, digestive vesicle; H, haemosomes; yellow arrows point towards contact sites of haemosomes and digestive vesicles. Scale bar indicates 500 nm. (*g*) RT-qPCR analysis of *ir-gst1* in adult female midguts dissected at 3 days of feeding and AD (FE—fully engorged ticks); each dataset is derived from RNA extracts of three individual females.

During blood-feeding, ticks internalize haemoglobin in their midgut cells, where it is hydrolysed under the acidic conditions of the endo-lysosomal vesicles by a network of mainly cysteine proteases [4]. Endocytosed host haemoglobin becomes associated with large endocytic vesicles ranging from 3–12 µm in *Rhipicephalus microplus* [29] and less than 1 µm in *I. ricinus* ticks [30]. Haem is then released from digested haemoglobin and accumulates in lysosomal residual bodies, also referred to as haemosomes [7,29]. From previous studies in other organisms, it is clear that HRG1 proteins localize to membranes of vesicles of the endo-lysosomal system [16,31], which is in line with predicted (figure 1*b*) and documented (figure 3*c*) targeting of *Ir*HRG. To assess if *Ir*HRG also participates in haem trafficking within the endo-lysosomal network of the tick digest cells, we followed haem accumulation in semi-thin (figure 6*a,b*) and ultra-thin (figure 6*c,d*) sections of tick midgut cells under light microscopy and transmission electron microscopy (TEM), respectively. Tick midgut digest cells of *Ir*HRG-KD ticks formed significantly larger haem-dense vesicles (haemosomes) than those of control ticks (figure 6*e*). In both cases, haemosomes were found to be associated with digestive vesicles, as identified using anti-cathepsin B-specific antibodies [32] (figure 6*f*). We have previously characterized a haem-binding glutathione S-transferase (*Ir*GST1) that is specifically expressed in the midgut of blood-fed and not serum-fed *I. ricinus* ticks [19,33]. Transcription of the *ir-gst1* gene is induced by the availability of dietary haem in the cytosol of digestive cells [33]. To examine whether *Ir*HRG facilitates *ir-gst1* expression, we performed RNAi-silencing of *Ir*HRG and found that *ir-gst1* mRNA levels were significantly reduced in *Ir*HRG-KD ticks compared to the GFP controls (figure 6*g*). This result confirmed that haem bioavailability within tick midgut digest cells was mediated by *Ir*HRG haem transport.

On the organism level, however, *Ir*HRG-KD ticks displayed the comparable capacity to fully engorge and reproduce when fed *in vivo* on full blood [34] (electronic supplementary material, figure S6A–C), as well as when fed *in vitro* low-haemoglobin-containing serum or serum alone (electronic supplementary material, figure S6D–F), indicating high redundancy of a haem transport system in tick tissues.

## 3. Discussion

Many species of arthropod lineages do not appear to code for HRG homologues. Using BlastP homology searches, we detected HRG homologues in ants, shrimps and ticks, but unexpectedly, no HRG homologue was found in blood-feeding insects. Admittedly, sequence similarity between identified homologues within arthropods or nematodes was very low. Therefore, we cannot rule out the presence of a functional homologue with very distant primary sequence beyond the identification constraints of the BlastP search. In this work, we took a synergistic approach of genetics, biochemistry, and microbiology to elucidate the function of *Ir*HRG in the hard tick *I. ricinus*, the European vector for Lyme borreliosis and tick-borne encephalitis.

Apart from its canonical functioning as an enzymatic cofactor, haem also serves as a signalling molecule regulating the expression of gene transcripts [35]. We have previously described positive regulatory mechanisms in the tick midgut, which were mediated by iron or haem signalling [3,19,33,36]. By contrast, SLC48A1 protein was first discovered through microarray screens in *C. elegans* as a gene with repressed transcription in response to high haem availability in the medium and was denoted as a HRG [16,25]. Here, we showed that *Ir*HRG transcript levels were not elevated during tick feeding of a haem(oglobin)-depleted diet (i.e. serum feeding). As the haem content in the *C. elegans* diet is not predictable, regulatory mechanisms need to be employed to secure haem acquisition. Ticks, in contrast, are obligatory blood-feeders in all developmental stages, i.e. haemoglobin is a staple constituent of every tick diet. This means that ticks would not face a shortage of haem during feeding. Therefore, a signalling mechanism promoting *Ir*HRG expression as a means to increase haem acquisition (negative regulation) cannot and does not exist. The tick *Ir*HRG (HRG), is, in fact, likely haem-non-responsive. Having said that, we cannot achieve a zero haem diet, as we have not yet succeeded in feeding ticks on a chemically formulated diet that would allow omitting haemoglobin altogether, and thus we could not avoid having traces of haemoglobin in blood serum (accounting for 6–10 µM haem, electronic supplementary material, figure S6). The responsiveness of HRG to haem in unicellular parasites does not seem to be universal. The *Trypanosoma brucei tb*-*hrg* homologue, which is mainly expressed in the insect stages rather than bloodstream forms infecting vertebrates, was not responsive to exogenous haemin in the insect stages [12,13]. By contrast, the insect stage of another kinetoplastid parasite, *Leishmania amazonensis,* responds to haemin-replete conditions in the culture by suppressing mRNA levels of its HRG homologue [11], a similar mechanism to that of *C. elegans* [16]. An HRG homologue of *Trypanosoma cruzi* (*Tc*HRG) responds to the presence of haem and decreases the transcript and protein level in the epimastigotes of the parasite [37]. It is noteworthy that experimental set-ups using diverse organisms with very complex life cycles cannot be compared and may not be aiming at the informative developmental stages where regulation presumably takes place, so might be over-looked or really be non-existent.

Expression of *Ir*HRG in *hem1Δ* yeasts enhanced exogenous haem-mediated cellular proliferation (figure 3), similarly to HRGs from other metazoan parasites [14,15], as well as enhancing catalase activity. Supplementation of yeast medium with haemoglobin induced higher catalase activity than equimolar haem supplementation in HRG-expressing yeast cells (electronic supplementary material, figure S3). We speculate that protein-bound haem, in the form of haemoglobin, is more bioavailable as it is taken up by endocytosis and trafficked to the digestive vacuole where the liberated haem is then efficiently imported into the cytosol by highly abundant *Ir*HRG at the organelle membrane. By contrast, haem not complexed with a protein vehicle might be prone to polymerization [38], decreasing its specific cellular uptake. Altogether, our yeast-based data supports the concept of *Ir*HRG aiding translocation of exogenous haem across (sub)cellular membranes inside the cell.

RNAi-mediated silencing was employed to elucidate *Ir*HRG function in ticks. Using light, fluorescent and electron microscopy, we visualized the accumulation of haem or haem analogue signals in vesicles of tick digest cells. Interestingly, we did not observe reduction of haemosome size upon RNAi as demonstrated for another haem transporter [39], but in fact the opposite, i.e. an increase of haemosome size. We thus speculate that haemosomes are formed either upon association with digestive vesicles or represent their terminal stage. One way or another, haem aggregates in vesicles where proteases are no longer present, indicating export from digestive vesicles. We argue that immediate diversion of liberated haem from the digestive vesicles into haemosomes prevents haem-mediated inhibition of acidic proteases, similar to a mechanism reported for *Plasmodium* spp. [40]. In contrast with ticks, however, haemozoin forms within the site of haemoglobin digestion, i.e. the digestive vacuole of *Plasmodium* spp. [41,42]. Our results further suggest that haem is expelled from digestive vesicles through physical association with destined haemosomes. Enlargement of haemosomes in *Ir*HRG-KD ticks can be interpreted in a way that *Ir*HRG, likely localized to the haemosome membrane, functions as regulatory vent controlling the release of haem to the cytosol. This hypothesis presents haemosomes as a sink of liberated haem, which is constantly imported from digestive vesicles, and then actively regulates cellular bioavailability of haem in midgut digest cells through *Ir*HRG-mediated export, and retains excess haem as an aggregate within the haemosomes. Certainly, highly specific anti-*Ir*HRG antibodies are needed to undeniably confirm the protein localization. This will further help us disclose whether haem is released to the cytosol directly from the digestive vesicles, through haemosomes, or both.

From an applied point of view, blocking potential haem trafficking by *Ir*HRG silencing did not result in reduced viability or developmental effects in blood-fed or low-haemoglobin serum-fed ticks (electronic supplementary material, figure S6). Loss-of-function (knock-downs/knock-outs) data from unicellular parasites mostly resulted in a compromised content of imported haem and reduced cellular viability. While the *hrg*-KD in the bloodstream form of *T. brucei* resulted in growth arrest and morphological alterations [12], the *hrg-*KD of *T. brucei* procyclics did not result in any reduced growth phenotype under standard culture conditions unless challenged with low haemin conditions; this significantly reduced the proliferation rate of the parasite [13]. *L. major* single knock-out promastigotes clearly contained lower levels of haem or ZnPPIX compared to the control, and HRG add-backs restored the take-up levels of porphyrins [12]. *L. amazonensis hrg*-single knock-out parasites replicated more slowly in a haemin-poor medium [24]. Similarly to unicellular parasites [43], ticks code for haem transporters of different protein families. For example, the adenosine triphosphate (ATP)-binding cassette (ABC) transporter ABCB10, identified in *R. microplus* (GenBank accession number JN098446.1), was shown to be a haem transporter [39]. Unlike previously described haem transporters of the ABC protein family localized to mitochondrial membranes [44], the tick midgut ABCB10 is likely located in the membrane of the digestive vesicles. Its *I. ricinus* orthologue (GenBank accession number GANP01009746.1) [45] thus might compensate for reduced levels of HRG in respective haem transporting pathways. The lack of apparent feeding phenotype in *Ir*HRG-KDs negates the potential of this protein to be an effective drug target [34], as it might do for unicellular parasites [1,46].

Enzymes or proteins that use haem as a cofactor may insert GaPPIX in their catalytic centres instead of haem, rendering the enzymes inactive. As gallium has only one valence state, Ga^3+^, it cannot be oxidized or reduced and is thus unable to substitute for Fe in biological reactions [47]. In *Pseudomonas aeruginosa*, GaPPIX inhibits its aerobic growth by targeting cytochromes, thus interfering with cellular respiration [48]. Blood-feeding of ticks, which likely saturates haem-binding proteins with acquired haem, thereby preventing GaPPIX from exerting its toxicity, allowed ticks to reach comparable engorged weights irrespective of the levels of GaPPIX in their blood meal. By contrast, serum feeding, which likely leaves the network of haem-binding proteins in their *apo*-states (haem-free), and, therefore able to form complexes with toxic GaPPIX, results in GaPPIX-mediated toxicity. GaPPIX toxicity was also conditioned by the absence of exogenous haem in *Brugia malayi* [14]. We show that GaPPIX-mediated toxicity is displayed in ticks by feeding engorgement arrest, which can be partially reversed by RNAi-mediated silencing of *Ir*HRG. We argue that the achieved resistance to GaPPIX-mediated toxicity in *Ir*HRG-KD ticks is due to reduction of GaPPIX translocation from the digestive vesicular system of tick midgut digest cells.

Our combined data demonstrate that the tick homologue *Ir*HRG is the *bona fide* tetrapyrrole transporter of FePPIX, ZnPPIX and GaPPIX, with physiological relevance to ticks for haem mobilization, i.e. converting exogenous haem into endogenous haem by its translocation from digestive vesicles, possibly via haemosomes, in order to fuel the intracellular haem-driven enzymatic network.

## 4. Material and methods

### 4.1. Tick maintenance and natural feeding

Adult *I. ricinus* ticks were collected in the forest near České Budějovice. Ticks were kept at 24°C and 95% humidity under a 15 : 9 h day/night regime. All laboratory animals were treated in accordance with the Animal Protection Law of the Czech Republic no. 246/1992 Sb., ethics approval no. 25/2018. The study was approved by the Institute of Parasitology, Biology Centre of the Czech Academy of Sciences (CAS) and Central Committee for Animal Welfare, Czech Republic (protocol no. 1/2015).

### 4.2. Tick membrane feeding

Membrane feeding of ticks was performed using a six-well plate format according to Kröber and Guerin [49]. Whole bovine blood was collected in a local slaughter house and manually defibrinated. To obtain serum, whole blood samples were centrifuged at 2500*g*, 10 min, 4°C and the resulting supernatant was collected and centrifuged again at 10 000*g*, 10 min, 4°C. Red blood cells were received as a pellet and were washed three times in sterile phosphate-buffered saline (PBS). Fifteen ticks were placed in a feeding unit lined with a thin (approximately 120 µm for adults; approximately 80 µm for juveniles) silicone membrane. After 24 h, unattached or dead ticks were removed and an equal number of males were added to the attached females in the feeding unit of adult ticks. Diets were exchanged in a 12 h regime, with concomitant addition of 1 mM ATP and gentamicin (5 µg ml^−1^). For diet supplementation, bovine haemoglobin (Sigma H2500) or haemin (Sigma H9039) were used. For membrane feeding experiments, the haemin stock solution (5 mM haemin dissolved in 100 mM NaOH) was diluted to 625 µM final concentration, establishing haem equimolarity with 1% haemoglobin (w/v). GaPPIX (Frontier Scientific P40167) was dissolved in 100 mM NaOH as a 5 mM stock.

### 4.3. Growth assay of *alas*^−^ deficient yeast strain and catalase assay

*Ir*HRG was amplified from tick tissue-specific cDNA using full-sequence specific primers (electronic supplementary material, table S2). The insert was further sub-cloned into destination vector: pDR ccdB-GFP-ura3 by Gateway™ LR Clonase™ II Enzyme mix (ThermoFisher Scientific 11791020). The cloning reaction was transformed into chemically competent DH5*α E. coli* cells (ThermoFisher Scientific), which were selectively grown on LB^KAN^ plates. Miniprep plasmid preparations were then obtained from LB^KAN^ liquid cultures to confirm the sequence of the insert.

*S. cerevisiae* BY4742 wild-type and *hem1Δ* (BY4742*hem1Δ*:BY4742,MAT *α*;his3*Δ*1;leu2*Δ*0;lys2*Δ*8;ura3*Δ*0;hem1::LEU2) were grown at 30°C in YPD (yeast extract–peptone–dextrose) or appropriate synthetic complete (SC) medium supplemented with 250 µM ALA hydrochloride, haemin (Sigma) or GaPPIX (Frontier Scientific). Cells were grown in YPD + ALA and were transformed with pDR experimental or control plasmid through the lithium acetate protocol [50], and transformants were selected on 2% w/v glucose SC^−ura^ + ALA plates.

Catalase activity was measured as described [51], but with some modifications. Briefly, yeast cells were cultured for 24 h without ALA to deplete haem and then incubated for 16 h in the absence or presence of either 0.5 µM bovine haemoglobin or 2 µM haemin. Washed yeast cells were then adjusted to a A_600_ of 5 in 1 ml of H_2_O. Three hundred microlitres of 1% Triton X-100 and 300 µl of 30% H_2_O_2_ were then added. Catalase-generated oxygen bubbles were trapped by Triton X-100 and observed as a foam, the height of which was proportional to enzyme activity.

### 4.4. Fluorescent microscopy and image processing (yeast cells)

The *haem-1Δ* yeast strains expressing *Ir*HRG_GFP, *Lm*HRG_GFP or GFP were used in the exponential growth phase. Yeast cells were washed twice in 500 µl of PBS, fixed with 2% paraformaldehyde and processed by microscopic observation. Images of yeast cells were acquired using an Olympus epifluorescence microscope and further processed using FIJI software (http://www.fiji.sc/Fiji).

### 4.5. Midgut micro-dissection, RNA extraction, cDNA synthesis and RT-qPCR

Membrane-fed *I. ricinus* larvae, nymphs and females were removed from the membrane or collected at the fully engorged state. Fully engorged larvae and nymphs were homogenized using a micro pestle in RA1 buffer (Macherey-Nagel, Germany) supplemented with β-mercaptoethanol. Midguts and ovaries of adult females were dissected under a drop of ice-cold DEPC-treated PBS and then homogenized in RA1 buffer supplemented with β-mercaptoethanol using a 29 G syringe. Total RNA was isolated from dissected tissues (intact or partial tissue from one individual was used per isolation) and whole body (seven larvae and a single nymph was used per isolation) homogenates using a NucleoSpinRNA II kit (Macherey-Nagel, Germany) and stored at −80°C. Prior to cDNA synthesis, RNA integrity was examined by agarose gel electrophoresis. cDNA preparations were made from 0.2 µg (juvenile ticks) or 0.5 µg (tissues of adult ticks) of total RNA in independent triplicates using the transcriptor high-fidelity cDNA synthesis kit (Roche Diagnostics, Germany). The cDNAs served as templates for subsequent quantitative expression analysis by RT-qPCR using the Fast Start Universal SYBR Green Master Kit (Roche). Samples were run on a LightCycler 480 (Roche) and relative expression was analysed by the ΔΔCt method. Expression profiles were normalized to *I. ricinus* elongation factor 1*α* (*ef*-*1α*) [52]. A list of primers is available as electronic supplementary material, table S2.

### 4.6. Phylogenetic analysis

The protein sequence obtained was verified by NCBI-BLAST search with the closest scores being to the *I. scapularis* and *A. variegatum* protein sequences. The dataset for following phylogenetic analysis was composed from available sequences in GenBank and UniProt to equally represent all the main groups of vertebrates, insects, nematodes and protists, among which the HRG transporter was identified. Sequences were aligned using the MAFFT software version 7 [53], and the final alignment was manually edited by Geneious Prime software 2019.1.1. (https://www.geneious.com). The best-fit model of molecular evolution was selected using akaike information criteria (AIC) in the ProtTest 3 software [54]. ML was performed under the empirical mixture model of substitution CAT-LG + G chosen by AIC. ML analyses were computed by the W-IQ-Tree software [55]. The reliability of branching patterns within the ML tree was tested by bootstrapping with 1000 resamplings, and the analyses were run independently and repeated three times. The final tree was visualized and rooted in the FigTree software v. 1.4.4 [56]. The protein (*Ir*HRG) sequence was deposited in GenBank/UniProt under accession number A0A131XSI2. Nei's genetic distances between sequences were calculated in Geneious.

### 4.7. ZnPPIX fluorescent microscopy

Uptake of a fluorescent metalloporphyrin, zinc mesoporphyrin, as a fluorescent haem analogue, was used to characterize haem intracellular pathways similarly to a previously published protocol [39]. A 20 mM stock solution of ZnPPIX (SigmaAldrich 691550-M) was dissolved in 100 mM NaOH and further diluted in sterile PBS before serving to ticks. Five microlitres of 100 µM ZnPPIX were introduced (2 h at 37°C) through 10 µl microcapillaries (Sigma-Aldrich P0674) into *I. ricinus* ticks, pre-fed 5 days on a guinea pig. Ticks were then rested for 24 h at 21°C. ZnPPIX was visualized in the dissected midguts of partially engorged ticks subjected as described below to RNA interference. The fluorescent images were obtained using a fluorescence microscope Olympus BX63 (filter U-FGWA, emission 575–625 nm) and DP74 digital camera.

### 4.8. Toluidine blue staining and transmission electron microscopy

Adult unfed females were injected with *Ir*HRG or *gfp* dsRNA (see below) and fed on guinea pigs for 5 days. The gut tissue was dissected and transferred to a fixative (2.5% glutaraldehyde) and incubated at 4°C, overnight. Samples were further washed with 0.1 M Na-phosphate buffer (PB) and incubated with 2% osmium tetroxide for 2 h at room temperature (RT), and washed again with PB. Tissues were dehydrated in ascendant acetone dilutions (30%, 50%, 70%, 80%, 90%, 95% and 100% for 15 min at each step). The samples were infiltrated stepwise in acetone mixed with EMbed 812 epon resin (EMS) (acetone : epon ratios of 2 : 1, 1 : 1, 1 : 2, at RT, for 1.5 h at each step followed by 100% Epon resin overnight). Embedded samples were thermo-polymerized at 62°C for 48 h. Semi-thin sections (400 nm) were stained with toluidine blue for 1 min at 60°C.

Ultra-thin sections (90 nm) were picked up on copper grids, contrasted in ethanolic uranyl acetate for 30 min and in lead citrate for 20 min. Semi-thin sections were observed under the Olympus BX53 light microscope and ultra-thin sections were observed in a JEOL 1010 transmission electron microscope.

### 4.9. High-pressure freezing and immuno-gold labelling

Guts were dissected from semi-engorged ticks. A piece of gut was transferred to a copper carrier in 20% bovine serum albumin (BSA) in PBS and immediately inserted into the rapid transfer system of the EM PACT2 (Leica) and frozen at a pressure of 2.1 kbar. The frozen samples were transferred under liquid nitrogen to cryovials with anhydrous acetone containing 0.25% glutaraldehyde and 0.25% uranylacetate. For freeze substitution, cryovials were placed in the chamber of a Leica AFS freeze substitution system/device (Leica EM AFS) precooled to −90°C. Samples were maintained 96 h at −90°C. Freeze-substituted samples were then allowed to warm to −20°C at a rate of 5°C/h. After 23 h were samples allowed to warm again at a rate of 5°C/h to −10°C. At −10°C were specimens washed three times for 1 h in fresh anhydrous acetone. After the washes, samples were gradually infiltrated with LRW resin (London Resin Company Ltd) diluted in acetone at −10°C (in ratios of 10% and 40% for 1 h and 60% and 80% for 2 h and in 100% resin for 2 h and overnight). After infiltration, LR white was polymerized under UV light for 48 h at −10°C in the FS unit. Ultra-thin sections (90 nm in thickness) were cut on a Leica ultramicrotome and placed on formvar-coated copper grids. Sections were blocked in 1% FSG (fish skin gelatine) in PBS + 0.1 M glycine for 1 h and subsequently exposed to antigen-purified primary antibody (cathepsin B, 1 : 5 in blocking solution) for 3 h. Grids were washed 3 × 1% FSG in PBS + 0.1 M glycine for 3 min and incubated with secondary antibody (goat anti-rabbit, 1 : 40) conjugated to gold particles of 10 nm in diameter for 1 h. In the end, all sections were washed again several times with blocking solution and dH_2_O. After immunolabelling, sections were contrasted in ethanolic uranyl acetate for 5 min, lead citrate for 3 min and observed in a JEOL 1010 transmission electron microscope.

### 4.10. RNA interference

dsRNA was designed as before [34], and synthesized using the MEGAscript T7 transcription kit (Ambion) according to the previously described protocol [34]. *I. ricinus* females were injected into the haemocoel through to the coxae with *Ir*HRG-specific dsRNA or control *gfp* dsRNA (0.4 µl; 3 µg µl^−1^) using a Narishige microinjector, allowed to rest for 1 day and then fed naturally on guinea pigs or on the artificial membrane feeding system. The efficiency of RNA-mediated silencing of *Ir*HRG gene expression was verified by RT-qPCR in tick midguts (figure 5*a*) and ovaries (electronic supplementary material, figure S7). Fully engorged weights were recorded, and embryos in oviposited eggs were visualized by light microscopy identically to our previously established protocol [3].

## Data accessibility

This article has no additional data.

## References

[1] Perner J, Gasser RB, Oliveira PL, Kopacek P. 2019 Haem biology in metazoan parasites — ‘the bright side of haem’. Trends Parasitol. **35**, 213-225. (10.1016/j.pt.2019.01.001) 30686614

[2] Gracasouza A, Mayamonteiro C, Paivasilva G, Braz G, Paes M, Sorgine M, Oliveira M, Oliveira P. 2006 Adaptations against heme toxicity in blood-feeding arthropods. Insect Biochem. Mol. Biol. **36**, 322-335. (10.1016/j.ibmb.2006.01.009) 16551546

[3] Perner J, Sobotka R, Sima R, Konvickova J, Sojka D, Oliveira PL, Hajdusek O, Kopacek P. 2016 Acquisition of exogenous haem is essential for tick reproduction. eLife **5**, e12318. (10.7554/eLife.12318) 26949258 PMC4821805

[4] Sojka D et al*.* 2013 New insights into the machinery of blood digestion by ticks. Trends Parasitol. **29**, 276-285. (10.1016/j.pt.2013.04.002) 23664173

[5] Agbede RIS. 1986 Scanning electron microscopy of digest cells in the midgut epithelium of *Boophilus microplus*. Exp. Appl. Acarol. **2**, 329-335. (10.1007/bf01193899) 3451868

[6] Tarnowski BI, Coons LB. 1989 Ultrastructure of the midgut and blood meal digestion in the adult tick *Dermacentor variabilis*. Exp. Appl. Acarol. **6**, 263-289. (10.1007/bf01193300) 2743838

[7] Lara FA et al. 2003 A new intracellular pathway of haem detoxification in the midgut of the cattle tick *Boophilus microplus*: aggregation inside a specialized organelle, the hemosome. J. Exp. Biol. **206**, 1707-1715. (10.1242/jeb.00334) 12682102

[8] Thöny-Meyer L. 2009 Heme transport and incorporation into proteins. In Tetrapyrroles. Molecular biology intelligence unit. New York, NY: Springer.

[9] Chambers IG, Willoughby MM, Hamza I, Reddi AR. 2021 One ring to bring them all and in the darkness bind them: the trafficking of heme without deliverers. Biochim. et Biophys. Acta (BBA) **1868**, 118881. (10.1016/j.bbamcr.2020.118881) PMC7756907 33022276

[10] Reddi AR, Hamza I. 2016 Heme mobilization in animals: a metallolipid's journey. Accounts Chem. Res. **49**, 1104-1110. (10.1021/acs.accounts.5b00553) PMC5629413 27254265

[11] Huynh C, Yuan X, Miguel DC, Renberg RL, Protchenko O, Philpott CC, Hamza I, Andrews NW. 2012 Heme uptake by *Leishmania amazonensis* is mediated by the transmembrane protein LHR1. PLoS Pathogens **8**, e1002795. (10.1371/journal.ppat.1002795) 22807677 PMC3395602

[12] Cabello-Donayre M et al*.* 2016 Trypanosomatid parasites rescue heme from endocytosed hemoglobin through lysosomal HRG transporters. Mol. Microbiol. **101**, 895-908. (10.1111/mmi.13430) 27328668

[13] Horakova E et al*.* 2017 The *Trypanosoma brucei Tb*Hrg protein is a heme transporter involved in the regulation of stage-specific morphological transitions. J. Biol. Chem. **292**, 6998-7010. (10.1074/jbc.M116.762997) 28232490 PMC5409468

[14] Luck AN, Yuan X, Voronin D, Slatko BE, Hamza I, Foster JM. 2016 Heme acquisition in the parasitic filarial nematode *Brugia malayi*. FASEB J. **30**, 3501-3514. (10.1096/fj.201600603R) 27363426 PMC5024691

[15] Toh SQ, Gobert GN, Malagon Martinez D, Jones MK. 2015 Haem uptake is essential for egg production in the haematophagous blood fluke of humans, *Schistosoma mansoni*. FEBS J. **282**, 3632-3646. (10.1111/febs.13368) 26153121

[16] Rajagopal A et al*.* 2008 Haem homeostasis is regulated by the conserved and concerted functions of HRG-1 proteins. Nature **453**, 1127-1131. (10.1038/nature06934) 18418376 PMC4058867

[17] Pek RH et al*.* 2019 Hemozoin produced by mammals confers heme tolerance. eLife **8**, e49503. (10.7554/eLife.49503) 31571584 PMC6773446

[18] White C et al*.* 2013 HRG1 is essential for heme transport from the phagolysosome of macrophages during erythrophagocytosis. Cell Metabolism **17**, 261-270. (10.1016/j.cmet.2013.01.005) 23395172 PMC3582031

[19] Perner J et al*.* 2016 RNA-seq analyses of the midgut from blood- and serum-fed *Ixodes ricinus* ticks. Sci. Rep. **6**, 36695. (10.1038/srep36695) 27824139 PMC5099782

[20] Yuan X, Protchenko O, Philpott CC, Hamza I. 2012 Topologically conserved residues direct heme transport in HRG-1-related proteins. J. Biol. Chem. **287**, 4914-4924. (10.1074/jbc.M111.326785) 22174408 PMC3281596

[21] Misra S, Puertollano R, Kato Y, Bonifacino JS, Hurley JH. 2002 Structural basis for acidic-cluster-dileucine sorting-signal recognition by VHS domains. Nature **415**, 933-937. (10.1038/415933a) 11859375

[22] Shiba T et al*.* 2002 Structural basis for recognition of acidic-cluster dileucine sequence by GGA1. Nature **415**, 937-941. (10.1038/415937a) 11859376

[23] Staudt C, Puissant E, Boonen M. 2016 Subcellular trafficking of mammalian lysosomal proteins: an extended view. Int. J. Mol. Sci. **18**, 47. (10.3390/ijms18010047) 28036022 PMC5297682

[24] Miguel DC, Flannery AR, Mittra B, Andrews NW. 2013 Heme uptake mediated by LHR1 is essential for *Leishmania amazonensis* virulence. Infection Immunity **81**, 3620-3626. (10.1128/IAI.00687-13) 23876801 PMC3811768

[25] Severance S, Rajagopal A, Rao AU, Cerqueira GC, Mitreva M, El-Sayed NM, Krause M, Hamza I. 2010 Genome-wide analysis reveals novel genes essential for heme homeostasis in *Caenorhabditis elegans*. PLoS Genetics **6**, e1001044. (10.1371/journal.pgen.1001044) 20686661 PMC2912396

[26] Protchenko O, Shakoury-Elizeh M, Keane P, Storey J, Androphy R, Philpott CC. 2008 Role of PUG1 in inducible porphyrin and heme transport in *Saccharomyces cerevisiae*. Eukaryotic Cell **7**, 859-871. (10.1128/ec.00414-07) 18326586 PMC2394968

[27] Zhang J, Chambers I, Yun S, Phillips J, Krause M, Hamza I. 2018 Hrg1 promotes heme-iron recycling during hemolysis in the zebrafish kidney. PLoS Genetics **14**, e1007665. (10.1371/journal.pgen.1007665) 30248094 PMC6171960

[28] Richter K, Van den Driessche F, Coenye T. 2017 Innovative approaches to treat *Staphylococcus aureus* biofilm-related infections. Essays Biochem. **61**, 61-70. (10.1042/ebc20160056) 28258230

[29] Lara FA. 2005 Tracing heme in a living cell: hemoglobin degradation and heme traffic in digest cells of the cattle tick *Boophilus microplus*. J. Exp. Biol. **208**, 3093-3101. (10.1242/jeb.01749) 16081607

[30] Sojka D et al*.* 2016 Multienzyme degradation of host serum albumin in ticks. Ticks Tick-borne Dis. **7**, 604-613. (10.1016/j.ttbdis.2015.12.014) 26724897

[31] O'Callaghan KM, Ayllon V, O'keeffe J, Wang Y, Cox OT, Loughran G, Forgac M, O'connor R. 2010 Heme-binding protein HRG-1 is induced by insulin-like growth factor I and associates with the vacuolar H^+^-ATPase to control endosomal pH and receptor trafficking. J. Biol. Chem. **285**, 381-391. (10.1074/jbc.M109.063248) 19875448 PMC2805445

[32] Franta Z et al*.* 2010 Dynamics of digestive proteolytic system during blood feeding of the hard tick *Ixodes ricinus*. Parasit. Vectors **3**, 119. (10.1186/1756-3305-3-119) 21156061 PMC3016361

[33] Perner J et al*.* 2018 Inducible glutathione S-transferase (*Ir*GST1) from the tick *Ixodes ricinus* is a haem-binding protein. Insect Biochem. Mol. Biol. **95**, 44-54. (10.1016/j.ibmb.2018.02.002) 29526768

[34] Hajdusek O, Sima R, Perner J, Loosova G, Harcubova A, Kopacek P. 2016 Tick iron and heme metabolism — new target for an anti-tick intervention. Ticks Tick-borne Dis. **7**, 565-572. (10.1016/j.ttbdis.2016.01.006) 26810909

[35] Liao R et al*.* 2020 Discovering how heme controls genome function through heme-omics. Cell Rep. **31**, 107832. (10.1016/j.celrep.2020.107832) 32610133 PMC7382780

[36] Hajdusek O, Sojka D, Kopacek P, Buresova V, Franta Z, Sauman I, Winzerling J, Grubhoffer L. 2009 Knockdown of proteins involved in iron metabolism limits tick reproduction and development. Proc. Natl Acad. Sci. **106**, 1033-1038. (10.1073/pnas.0807961106) 19171899 PMC2633537

[37] Pagura L, Tevere E, Merli ML, Cricco JA. 2020 A new model for *Trypanosoma cruzi* heme homeostasis depends on modulation of *Tc*HTE protein expression. J. Biol. Chem. **295**, 13 202-13 212. (10.1074/jbc.RA120.014574) 32709751 PMC7504937

[38] Ponka P, Sheftel AD, English AM, Scott Bohle D, Garcia-Santos D. 2017 Do mammalian cells really need to export and import heme? Trends Biochem. Sci. **42**, 395-406. (10.1016/j.tibs.2017.01.006) 28254242

[39] Lara FA et al*.* 2015 ATP binding cassette transporter mediates both heme and pesticide detoxification in tick midgut cells. PLoS ONE **10**, e0134779. (10.1371/journal.pone.0134779) 26258982 PMC4530934

[40] Jagt DLV, Hunsaker LA, Campos NM. 1987 Comparison of proteases from chloroquine-sensitive and chloroquine-resistant strains of *Plasmodium falciparum*. Biochem. Pharmacol. **36**, 3285-3291. (10.1016/0006-2952(87)90646-0) 3311049

[41] Chugh M, Sundararaman V, Kumar S, Reddy VS, Siddiqui WA, Stuart KD, Malhotra P. 2013 Protein complex directs hemoglobin-to-hemozoin formation in *Plasmodium falciparum*. Proc. Natl Acad. Sci. **110**, 5392-5397. (10.1073/pnas.1218412110) 23471987 PMC3619337

[42] Noland GS, Briones N, Sullivan DJ. 2003 The shape and size of hemozoin crystals distinguishes diverse *Plasmodium* species. Mol. Biochem. Parasitol. **130**, 91-99. (10.1016/s0166-6851(03)00163-4) 12946845

[43] Cabello-Donayre M et al*.* 2019 *Leishmania* heme uptake involves *Lm*FLVCRb, a novel porphyrin transporter essential for the parasite. Cell. Mol. Life Sci. **77**, 1827-1845. (10.1007/s00018-019-03258-3) 31372684 PMC11104922

[44] Zutz A, Gompf S, Schägger H, Tampé R. 2009 Mitochondrial ABC proteins in health and disease. Biochim. et Biophys. Acta (BBA) **1787**, 681-690. (10.1016/j.bbabio.2009.02.009) 19248758

[45] Kotsyfakis M, Schwarz A, Erhart J, Ribeiro JM. 2015 Tissue- and time-dependent transcription in *Ixodes ricinus* salivary glands and midguts when blood feeding on the vertebrate host. Sci. Rep. **5**, 9103. (10.1038/srep09103) 25765539 PMC4357865

[46] Laranjeira-Silva MF, Hamza I, Pérez-Victoria JM. 2020 Iron and heme metabolism at the leishmania–host interface. Trends Parasitol. **36**, 279-289. (10.1016/j.pt.2019.12.010) 32005611 PMC7161743

[47] Stojiljkovic I, Kumar V, Srinivasan N. 1999 Non-iron metalloporphyrins: potent antibacterial compounds that exploit haem/Hb uptake systems of pathogenic bacteria. Mol. Microbiol. **31**, 429-442. (10.1046/j.1365-2958.1999.01175.x) 10027961

[48] Hijazi S, Visca P, Frangipani E. 2017 Gallium-protoporphyrin IX inhibits *Pseudomonas aeruginosa* growth by targeting cytochromes. Front. Cellular Infection Microbiol. **7**, 12, (10.3389/fcimb.2017.00012) PMC5266731 28184354

[49] Kröber T, Guerin PM. 2007 *In vitro* feeding assays for hard ticks. Trends Parasitol. **23**, 445-449. (10.1016/j.pt.2007.07.010) 17681859

[50] Ito H, Fukuda Y, Murata K, Kimura A. 1983 Transformation of intact yeast cells treated with alkali cations. J. Bacteriol. **153**, 163-168. (10.1128/jb.153.1.163-168.1983) 6336730 PMC217353

[51] Iwase T, Tajima A, Sugimoto S, Okuda K, Hironaka I, Kamata Y, Takada K, Mizunoe Y. 2013 A simple assay for measuring catalase activity: a visual approach. Sci. Rep. **3**, 03081. (10.1038/srep03081) PMC3812649 24170119

[52] Nijhof AM, Balk JA, Postigo M, Jongejan F. 2009 Selection of reference genes for quantitative RT-PCR studies in *Rhipicephalus* (*Boophilus*) *microplus* and *Rhipicephalus appendiculatus* ticks and determination of the expression profile of Bm86. BMC Mol. Biol. **10**, 112. (10.1186/1471-2199-10-112) 20040102 PMC2809063

[53] Katoh K, Standley DM. 2013 MAFFT multiple sequence alignment software version 7: improvements in performance and usability. Mol. Biol. Evol. **30**, 772-780. (10.1093/molbev/mst010) 23329690 PMC3603318

[54] Darriba D, Taboada GL, Doallo R, Posada D. 2011 ProtTest 3: fast selection of best-fit models of protein evolution. Bioinformatics **27**, 1164-1165. (10.1093/bioinformatics/btr088) 21335321 PMC5215816

[55] Trifinopoulos J, Nguyen LT, von Haeseler A, Minh BQ. 2016 W-IQ-TREE: a fast online phylogenetic tool for maximum likelihood analysis. Nucleic Acids Res. **44**, W232-W235. (10.1093/nar/gkw256) 27084950 PMC4987875

[56] Rambaut A. 2019 Figtree v1.4.4. See http://tree.bio.ed.ac.uk/software/figtree/ (accessed 5 Dec 2019).

